# Endothelial Function and Physical Exercise

**DOI:** 10.5935/abc.20180211

**Published:** 2018-10

**Authors:** Luana Urbano Pagan, Mariana Janini Gomes, Marina Politi Okoshi

**Affiliations:** Faculdade de Medicina de Botucatu - Universidade Estadual Paulista (UNESP), Botucatu, SP - Brazil

**Keywords:** Endothelium, Vascular/physiopathology, Nitric Oxide, Vascular Tonus, Cardiovascular Diseases, Exercise Therapy, Rats

The endothelium is considered an active and dynamic tissue with important properties such as maintenance of blood circulation, regulation of vascular tone, microvascular permeability, signaling, and vascular angiogenesis and inflammatory response.^1^ The endothelium allows the connection among components of the circulation and body systems. Endothelial cells produce and, depending on the stimulus received, release factors that lead to vascular smooth muscle cells contraction or relaxation.^2^ Vascular tone control by the endothelium is modulated by the production and release of mediators such as nitric oxide, prostacyclins, prostaglandins, thromboxane, angiotensin II, endothelin-1 and reactive oxygen species. Under physiological conditions, these factors are balanced. Imbalance in the production of substances by the endothelium leads to triggering and progression of several conditions and diseases such as ischemia, thrombosis, atherosclerosis, arterial hypertension, inflammation and tumor growth.^1^^,^^2^ Therefore, vascular endothelial dysfunction is an important pathophysiological factor in human diseases.^3^

Endothelial dysfunction is mainly characterized by changes in endothelial actions involving the reduction of vasodilation and the induction of a pro-inflammatory or prothrombotic state.^3^ Due to its clinical importance, endothelial dysfunction is considered an independent predictor of cardiovascular risk. In addition, it can also be observed in non-cardiovascular diseases, such as rheumatic and autoimmune diseases.^2^

Among the substances produced by the endothelium, nitric oxide stands out, being a potent modulator of vascular and cardiac function. Insufficient production of nitric oxide, such as in aging and in several diseases, may result in an increase in reactive oxygen species and blood pressure, and adversely affects the physical capacity and health in general.^2^

Physical exercises have been advocated for the promotion of health and the non-pharmacological treatment of cardiovascular diseases. Regular practice of exercises results in numerous health benefits, such as improvement in body composition, physical capacity, insulin resistance, endothelial function, arterial hypertension, antioxidant status, quality of life,^4^^-^^12^ and an important effect on the endothelial system. During its practice, increased blood flow and shear stress improve vascular homeostasis by reducing the production of reactive oxygen species, and increasing the availability of nitric oxide in the endothelium.^13^

Because endothelial function and physical exercise have an important interface with cardiovascular diseases, we consider the review of this area in articles recently published by the *Arquivos Brasileiros de Cardiologia* in the Basic and Experimental Research Area relevant. In this Editorial, we have commented on three articles that have been published in the last two years, and which are related to endothelial changes from physical exercise, both in healthy rats and in spontaneously hypertensive rats.

Mota et al.^14^ observed that a single resisted exercise session improves the endothelial function, and increases nitric oxide synthesis in both the endothelium and the smooth muscle layer of healthy rats. As a parameter of vascular reactivity, endothelium-dependent vasodilation in the mesenteric artery was evaluated. Exercise practice increased insulin-induced vasodilation. As vascular relaxation was abolished by the nitric oxide synthesis inhibitor, the methyl ester of L'NG-nitro-arginine (L-NAME), the importance of nitric oxide in the vasodilator response was enhanced. According to the authors, exercise stimulates factors that increase the production of nitric oxide, such as vascular distension, catecholamine release and intermittent hypoxia. The increase in nitric oxide production was dependent on the volume of exercise, which suggests that a greater demand of oxygen and nutrients is involved in the beneficial effects of exercise on the endothelium.

Similar results were observed in hypertensive rats.^15^ A single session of resisted exercise provided the activation of endothelial nitric oxide synthase (eNOS), increased acetylcholine-induced aortic relaxation, and decreased reactivity to phenylephrine. The response to phenylephrine was abolished by L-NAME. Therefore, data reinforce that, even in arterial hypertension, the improvement of the endothelial function induced by a single session of resisted exercise is associated with the increase of nitric oxide synthesis.

Beneficial results were also observed after a long period of exercise (one hour/day on treadmill, 5 days a week, 8 weeks) in healthy rats.^7^ Martinez et al.^7^ observed that exercise reduced the contractile response of the aorta to noradrenaline and increased the relaxation induced by acetylcholine. On the other hand, the accumulated exercise protocol (four periods of 15 minutes per day on treadmill, 5 times per week, 8 weeks) did not result in improvement of endothelial function. Consequently, it is believed that the beneficial effects on the induction of regulatory factors that improve endothelial function are linked to the time of exercise exposure.

These experimental studies suggest that the practice of physical exercises plays a relevant role in the treatment of endothelial dysfunction. However, additional studies are needed to establish the best type, intensity and duration of exercise, and to allow more efficient prescribing.
